# Development and Validation of the Pregnancy Guilt Assessment Scale (PGAS): A Specific Tool for Assessing Guilt in Pregnancy

**DOI:** 10.3390/healthcare13243241

**Published:** 2025-12-10

**Authors:** Octavio Luque-Reca, Cecilia Peñacoba, Patricia Catalá

**Affiliations:** Department of Psychology, Rey Juan Carlos University, 28922 Alcorcón, Madrid, Spain; octavio.luque@urjc.es (O.L.-R.);

**Keywords:** gestational guilt, psychometric validation, PGAS, factor analysis, perinatal mental health, Spanish population

## Abstract

**Background:** Gestational guilt is an understudied emotional experience that can affect maternal well-being and prenatal bonding. This study aimed to develop and validate the Pregnancy Guilt Assessment Scale (PGAS) in a sample of Spanish pregnant women, assessing its factorial structure, reliability, and validity. **Methods:** Four phases were conducted: (1) item generation through focus groups (*n* = 17) and cognitive interviews (*n* = 8); (2) expert content validation (*n* = 3); (3) exploratory factor analysis (EFA) in a pilot sample (*n* = 85); and (4) confirmatory factor analysis (CFA) and validity testing in an independent sample (*n* = 171). Additional measures included antenatal depression, prenatal distress, affect, self-esteem, social support, and dispositional guilt. Internal consistency, correlations, and multiple regressions assessed reliability and convergent and incremental validity. **Results:** The final 16-item PGAS showed a bifactor structure with a general factor and four dimensions: G-LSC (Lack of self-care), G-UEE (Unmet emotional expectations), G-SP (Social pressure), and G-CWR (Conflict with work role). The model demonstrated good fit (χ^2^ = 109.42, df = 88, *p* = 0.061; CFI = 0.974; TLI = 0.965; RMSEA = 0.069; SRMR = 0.030) and high reliability (α total = 0.96; ω = 0.98; subscales α = 0.90–0.94). PGAS scores correlated positively with dispositional guilt, negative affect, prenatal distress, and antenatal depression. In regressions, G-UEE uniquely predicted depression (β = 0.213, *p* = 0.002) and G-SP predicted distress (β = 0.303, *p* < 0.001). Women who had considered pregnancy termination scored higher on guilt (*p* < 0.001). **Conclusions:** The PGAS is a reliable and valid instrument for assessing pregnancy-related guilt in Spanish women, with potential relevance for perinatal mental health research and clinical practice, while future studies should evaluate its performance in other cultural settings.

## 1. Introduction

### 1.1. Background and Rationale

During pregnancy, women undergo multiple physical, emotional, and social changes [1]. This profoundly transformative period can be accompanied by ambivalent affective experiences such as joy, fear, anxiety, or guilt [2,3]. Among these, pregnancy guilt has scarcely been explored, despite its growing recognition in clinical settings and among pregnant women themselves.

Research on perinatal mental health has focused primarily on depression and anxiety, leaving other relevant affective phenomena in the background. However, guilt is not merely a secondary or derivative manifestation of these conditions. Unlike broader affective states such as anxiety or depressive mood, guilt is conceptualized as a moral emotion characterized by self-evaluation, perceived responsibility, and reparative tendencies, with distinctive cognitive (self-blame, appraisals of moral responsibility), emotional (remorse, self-reproach), and behavioral (need for reparation or self-correction) components [3,4,5,6,7]. These features have specific implications for maternal identity, prenatal bonding, and decision-making during pregnancy, and justify addressing guilt as a separate focus of study rather than subsuming it under generic emotional distress. Guilt during pregnancy has been identified in qualitative studies as a common emotional experience, linked to unmet expectations, comparisons with idealized models of motherhood [8,9], and perceived inappropriate decisions regarding fetal well-being or the maternal role [3,10].

Empirical evidence indicates that feelings of guilt in this context can act as an emotional risk factor, interfering with psychological well-being, affecting prenatal bonding, and generating dysfunctional thoughts that persist into the postpartum period [2,3,10]. Therefore, it is urgent to advance their understanding, evaluation, and intervention from a preventive perspective and with adapted emotional support.

### 1.2. Conceptualization of Guilt During Pregnancy

Gestational guilt is defined as a morally laden emotional experience that emerges when a pregnant woman perceives that she has violated a norm, expectation, or ideal (her own or social) related to her role as a mother [4,5]. Unlike dispositional guilt, which is part of a stable personality style, gestational guilt is a transitory but intense emotional state linked to the experience of pregnancy and its multiple demands [4].

This form of guilt can be differentiated from emotional phenomena such as anxiety or sadness, in that it is associated with negative self-evaluation, self-reproach, the desire for reparation, and emotional inhibition. Thus, beyond general distress, gestational guilt involves a specific moral appraisal (“I should have acted differently”) and a felt need to repair or compensate for the perceived failure. It is also distinguished by its normative and moralizing burden, frequently influenced by cultural messages about how motherhood should be experienced [4].

From a clinical perspective, it has been observed that high levels of gestational guilt can be associated with a higher risk of perinatal depression, lower self-esteem, body dissatisfaction, greater ambivalence about motherhood, and difficulties bonding with the baby [6,11]. At a social level, this emotional experience is often intensified by discourses that promote rigid and unattainable ideals of a “good mother,” reinforcing self-imposed demands and emotional isolation [3,8,9].

Gestational guilt can also be conceptually distinguished from related constructs such as maternal self-blame, shame, and perinatal anxiety. While self-blame involves attributing responsibility to oneself for perceived failures, it may remain at the level of a cognitive attribution, whereas gestational guilt also includes a motivational tendency toward reparation in response to perceived moral violations [3,4]. Shame, in contrast, implies a negative evaluation of the whole self (“I am a bad mother”), whereas guilt involves a negative evaluation of specific behaviors (“I did not care for my body adequately”) [5] and is more likely to motivate corrective or reparative action rather than withdrawal or hiding. Anxiety is predominantly future-oriented and focused on threat, uncertainty, or potential harm, whereas guilt is more retrospective and tied to perceived moral transgressions or unmet expectations [6] and centrally involves moral self-evaluation rather than anticipation of external danger. These distinctions further support the need for a dedicated measure of gestational guilt tailored to pregnancy-specific experiences [7].

### 1.3. Need for a Specific Measure of Guilt During Pregnancy

Existing guilt instruments, such as general trait guilt scales or measures focused on specific domains (e.g., maternal employment) [12], conceptualize guilt primarily as a dispositional characteristic and do not capture pregnancy-specific triggers, such as concerns about fetal well-being, prenatal health behaviors, or tensions between maternal and professional roles [3,4]. Likewise, widely used perinatal measures (e.g., the Prenatal Distress Questionnaire or the Edinburgh Postnatal Depression Scale) [13,14,15,16], include guilt only as a minor symptom within broader emotional or depressive domains, and therefore lack sensitivity to the moral, cultural, and identity-related aspects that characterize guilt in pregnancy [5,6]. These limitations highlight the need for an instrument specifically designed to assess gestational guilt, rather than relying on generic dispositional or perinatal measures.

To date, there are no validated instruments specifically for assessing guilt during pregnancy, which represents a limitation for both research and clinical practice, as it hinders the early identification of risk profiles and the planning of targeted interventions. One of the few studies that explicitly addresses this emotional experience [7] emphasizes the importance of systematically assessing pregnancy-related guilt and stresses that understanding the reasons that generate these feelings is essential to determine the type of support that pregnant women may need. The development of the PGAS is therefore a key step in filling this gap, providing a contextualized and clinically useful measure of gestational guilt.

Moreover, adapting general guilt or perinatal stress instruments would not adequately address the cultural norms, contextual demands, and identity-based tensions inherent in gestational guilt [4,5]. Existing tools lack pregnancy-specific content and show limited sensitivity to sociocultural variability, particularly in Spanish-speaking and Mediterranean contexts, where cultural expectations around motherhood and fetal responsibility are especially salient [2], and similar patterns have also been documented in other cultural contexts [3,10]. Given that current guilt scales have not been culturally adapted for Spanish pregnant women [12], a new instrument was required to ensure contextual, linguistic, and psychometric appropriateness [17].

### 1.4. Objectives of the Study

The aim of this study was to develop and validate a specific scale for assessing guilt during pregnancy in Spanish women. The objective was to construct an instrument with adequate psychometric properties that would allow for the identification and quantification of guilt in pregnant women, as well as to explore its factor structure, reliability, and convergent and incremental validity.

### 1.5. The Present Research

The present study addresses the development and initial validation of the Pregnancy Guilt Assessment Scale (PGAS), a measure designed to assess guilt experienced by Spanish women during pregnancy. The validation process followed four sequential phases combining qualitative and quantitative methods to ensure content and construct validity. First (phase 1), focus groups with pregnant women were used to explore guilt-related experiences, followed by item drafting by the research team and a subsequent cognitive review with another group of participants, resulting in the “initial draft version” of the PGAS. Next (phase 2), a panel of experts quantitatively evaluated item relevance and clarity using a Content Validity Index, producing the “post-cognitive debriefing version” of the PGAS. In the third phase (phase 3), a pilot sample of pregnant women completed this “expert-refined version” to examine its underlying factor structure and guide item refinement. Finally (phase 4), the “post-EFA version” of the PGAS was administered to an independent sample of pregnant women to replicate the factor structure and assess its reliability, convergent and incremental validity, and group differences. Table 1 summarizes the phases, objectives, procedures, and samples involved in this validation process.

## 2. Materials and Methods

All phases of this research were conducted in strict accordance with the Declaration of Helsinki and approved by the Ethics Committee of the Rey Juan Carlos University. Participation across all phases was entirely voluntary, and written informed consent was obtained from all participants before data collection.

### 2.1. Participants and Procedures

#### 2.1.1. Phase 1—Item Generation and Comprehension Review

*Focus Groups (n = 17)*

Seventeen pregnant women participated in this first phase of the research (M age = 34.52; SD = 5.01), recruited from two primary healthcare centers in Madrid, Spain. The inclusion criteria were: (1) being Spanish nationals, (2) pregnant (any trimester: first, second, or third), (3) low obstetric risk, and (4) no diagnosed psychiatric disorders. No dropouts occurred during this phase.

Regarding the procedure followed, sessions were conducted between November and December 2024, moderated by a psychologist with expertise in qualitative research and assisted by an observer who took field notes. Two focus groups were conducted: one comprised 11 pregnant women, and the other was a mixed group with 6 pregnant women and 6 postpartum women. As the broader research project aimed to develop two distinct guilt measures (one for pregnancy and another for the postpartum period), only contributions from pregnant participants were considered for item generation in this study. Following established recommendations [18,19,20], each meeting began with introductions, explanation of goals and procedures (including audio recording and confidentiality assurances), and participant self-presentations. Open-ended questions guided discussions on guilt-related experiences during pregnancy, encouraging free sharing while respecting turn-taking and differing views. To manage the larger size of the first focus group (*n* = 11) and to reduce the risk of dominance or loss of quieter voices, we used a moderator accompanied by an observer and active moderation techniques (e.g., directed prompts to quieter participants, structured turn rounds, and brief summarizing interventions) as recommended in recent methodological guidance [19,20].

Sessions lasted around two hours and were audio-recorded with participants’ permission. The assistant also took systematic field notes capturing non-verbal cues and interactional dynamics to complement the audio record [19]. Recordings were transcribed verbatim, ensuring each speaker was identified. The research team conducted a systematic thematic analysis, reading transcripts multiple times to gain familiarity with the data, highlighting relevant text segments, coding them, and progressively grouping similar codes into broader categories to capture recurring ideas and patterns. These categories were then used to generate the initial draft of the PGAS.

*Cognitive Debriefing (n = 8)*

A separate convenience sample of eight pregnant women participated in this phase, conducted in February 2025 (M age = 34.52, SD = 5.01). The same inclusion criteria were applied as in the focus groups (Spanish nationality, pregnant at any stage, low obstetric risk, and absence of diagnosed psychiatric conditions).

Concerning the procedure, participants were asked to independently review the post-cognitive debriefing version of the PGAS, developed from focus group findings and prior literature. They assessed each item for comprehensibility (ease of understanding) and relevance (importance for the construct of guilt in pregnancy), suggesting rewording when needed. Feedback was used to refine or remove ambiguous statements, resulting in a clearer and contextually appropriate version for the next phase (see Section 2.3 for further details on this process).

#### 2.1.2. Phase 2—Expert-Based Content Evaluation (*n* = 3)

Three Spanish experts in psychology participated in this phase (M age = 34.52, SD = 5.01; range = 20–45 years). Their professional backgrounds included psychometrics, perinatal psychology, and maternal health, ensuring complementary expertise for the evaluation process. Experts were contacted via professional networks and invited to independently review the *post-cognitive debriefing version of the PGAS*. For each item, they rated relevance on a 4-point scale and could provide written comments to refine wording, suggest additional content, or recommend removal of ambiguous or redundant items. These evaluations were later aggregated and analyzed to inform the assessment of content validity and guide further item refinement.

#### 2.1.3. Phase 3—Preliminary Examination of the Underlying Factor Structure (*n* = 85)

Eighty-five pregnant women participated in this phase of the research (M age = 34.72 years; SD = 4.89). Inclusion criteria were the same as in previous phases (Spanish nationality, pregnant at any stage, low obstetric risk, and absence of diagnosed psychiatric conditions). Most participants were Spanish nationals (91.8%), with a small proportion from other countries (1.2–2.4% each). A majority had no previous children (64.7%), while 15.3% had considered pregnancy termination and 11.8% reported obstetric complications. Educational attainment was high, with 59% holding university or master’s degrees, and most participants were employed full-time (64.7%). Annual gross income ranged mainly between €13,737 and €36,632 (42.4%), with 22.4% preferring not to respond. On average, women reported 7.29 h of sleep daily (SD = 1.11), with 79.4% describing their sleep as good or regular. Most pregnancies were planned (77.6%), conception took an average of 11.26 months, and the mean gestational age was 23.65 weeks.

Participants were recruited through Netquest, an online opt-in panel managed by a professional survey company specialized in social and health research. The company contacted eligible women via email and mobile notifications from its registered database, applying quotas to ensure variability in sociodemographic profiles (e.g., age, region of residence). After confirming eligibility, participants accessed a secure online platform to complete the survey. Following content evaluation by the expert panel, the expert-refined version of the PGAS was administered online using a 4-point Likert scale (1 = totally false, 4 = totally true). On average, completing the questionnaire took approximately 5 min. All survey items required a response to proceed, eliminating missing data. Responses were stored directly in a protected database for subsequent psychometric analyses.

#### 2.1.4. Phase 4—Factor Structure Replication, Reliability, Group Comparisons, and Validity Testing (*n* = 171)

A total of 171 pregnant women residing in Spain participated in this phase (M age = 34.25 years; SD = 4.98; range = 20–45 years). Most were Spanish nationals (87.7%), with a minority from Latin American and European countries, mainly Colombia (2.3%) and several others (1.2% each). The same inclusion criteria were applied as in previous phases. Most had no previous children (57.3%), while 31% had considered pregnancy termination and 19.3% reported obstetric complications. The sample showed high educational attainment (67.3% with university or master’s degrees) and stable employment (66.7% full-time). Average sleep duration was 7.56 h (SD = 1.35), with 74.2% reporting regular or good quality sleep. Pregnancies were mostly planned (72.5%), conception time averaged 7.40 months (SD = 9.94), and gestational age was 21.36 weeks (SD = 10.21). The vast majority were married or cohabiting with a partner.

Participants were recruited through the same online panel managed by Netquest. Eligible women from its panel database received an invitation to participate voluntarily and accessed a secure platform to complete the study. The survey included sociodemographic and pregnancy-related questions, the post-EFA version of the PGAS, and several validated self-report instruments designed to test the convergent, discriminant, and incremental validity of the scale. These assessed antenatal depressive symptoms, prenatal distress, affect, self-esteem, perceived social support, and dispositional guilt. All items were mandatory, preventing missing data, and the entire questionnaire took approximately 20 min to complete. Responses were stored automatically in a secure database for subsequent analyses.

### 2.2. Measures

The PGAS was the sole instrument used in phases 1 to 3; all subsequent measures were implemented exclusively in phase 4 together with the PGAS.

*Guilt in pregnancy*

Pregnancy-related guilt was assessed using the Pregnancy Guilt Assessment Scale (PGAS), a measure created for the purposes of this study. An initial pool of 30 items was generated from qualitative analyses and expert input. Instructions asked participants to indicate how frequently they experienced guilt associated with specific pregnancy-related thoughts and experiences in recent weeks, using a 6-point Likert scale ranging from 1 (never) to 6 (always). The final number of items and the factor structure resulting from the validation process are reported in the Section 3 (see Table 2).

*Antenatal depression*

To assess antenatal depressive symptomatology, we used the Edinburgh Postnatal Depression Scale (EPDS) [11], employing the Spanish version developed by García-Esteve et al. [16], which has been validated for use in pregnant women in Spain [21]. The EPDS consists of 10 items that evaluate mood symptoms experienced over the past 7 days. Each item is rated on a 4-point Likert scale ranging from 0 to 3, yielding a total score between 0 and 30, with higher scores indicating more severe depressive symptoms. The original Spanish validation reported adequate internal consistency (α = 0.79) [12]. In the present study, Cronbach’s α was 0.91.

*Prenatal distress*

The Prenatal Distress Questionnaire (PDQ) [14] is a self-report instrument designed to assess distress specifically related to pregnancy, including maternal concerns about their own physical and emotional well-being, relational changes, the health of the baby, and the process of delivery. It consists of 12 items rated on a 5-point Likert scale from 0 (not at all) to 4 (very much), with higher scores indicating greater prenatal distress. We used the Spanish version adapted and validated by Caparrós-González et al. [14], which demonstrated good internal consistency in pregnant women in Spain (α = 0.74). In our sample, the PDQ also showed good internal consistency (α = 0.81).

*Positive and negative affect*

To assess general affective states, we used the International Positive and Negative Affect Schedule–Short Form (I-PANAS-SF) [16], employing the Spanish version translated by Gargurevich [22]. This 10-item self-report instrument was derived from the original PANAS [23] and is designed to measure two broad dimensions of affect: positive affect (PA) and negative affect (NA). It comprises two subscales of five items each, rated on a 5-point Likert scale from 1 (very slightly or not at all) to 5 (extremely), with higher scores indicating greater intensity of the corresponding affective state. Previous research has reported adequate internal consistency for both the original and Spanish versions (α ≥ 0.72) [16,17]. In the present study, internal consistency was adequate, with α = 0.77 for PA and α = 0.83 for NA.

*Self-esteem*

To assess global self-esteem, we employed the Rosenberg Self-Esteem Scale (RSES) [19], using the Spanish adaptation developed by Martín-Albo et al. [24]. This unidimensional instrument evaluates overall self-worth through 10 items, five formulated positively and five negatively. Each item is rated on a 4-point Likert scale ranging from 1 (strongly disagree) to 4 (strongly agree), with higher scores indicating greater self-esteem. The Spanish version has demonstrated good psychometric properties in non-clinical adult samples (α = 0.85) [24]. In our sample of pregnant women, internal consistency was excellent (α = 0.90).

*Perceived social support*

Perceived social support was measured using the Multidimensional Scale of Perceived Social Support (MSPSS) [21], employing the Spanish version adapted by Landeta and Calvete [25]. The MSPSS assesses the extent individuals perceive support from three sources: family, friends, and a significant other. The original instrument consists of 12 items rated on a 7-point Likert scale ranging from 1 (very strongly disagree) to 7 (very strongly agree), yielding both subscale and total scores. Due to the overall length of the assessment protocol and to minimize participant burden, we selected 6 items from the original scale, ensuring representation from the three support dimensions. In the present study, the total perceived social support score was used. This ad hoc abbreviated version showed good internal consistency in our sample (α = 0.86).

*Dispositional guilt*

To evaluate the general tendency to feel unjustified or excessive guilt, we used the items corresponding to factor 2 of the *Escala de Sentimiento de Culpa [Guilt Feeling Scale]* (SC-35) [23], developed and validated in Spain. These five items specifically assess the tendency to experience guilt in situations where such feelings are unwarranted or disproportionate. Each item was rated on a 4-point Likert scale ranging from 1 (*totally false*) to 4 (*totally true*), with higher scores indicating greater dispositional guilt. In our sample, the internal consistency was high (α = 0.86).

### 2.3. Data Analysis

Quantitative analyses were performed using R (version 4.3.0; R Foundation for Statistical Computing, Vienna, Austria) with the packages psych, effsize, REdaS, EFAtools, e1071, lavaan, and haven. Qualitative analyses were conducted using NVivo 12 (QSR International Pty Ltd, Melbourne, Australia) to perform thematic coding of focus group data, complemented by manual review of participant ratings and comments during item evaluation procedures (i.e., cognitive debriefing and expert panel).

#### 2.3.1. Phase 1—Item Generation (Focus Groups and Cognitive Debriefing)

The focus groups, conducted following established guidelines [18,19,20], were analyzed in NVivo 12 using a thematic and inductive approach by two independent coders, who reached consensus on a set of themes describing recurrent experiences of guilt during pregnancy. A brief review of the literature on guilt and motherhood was used to complement these themes and ensure that relevant domains were not overlooked. Based on this material, and following established guidelines for scale development [26], the research team generated concise, pregnancy-specific items in language close to participants’ own expressions, aiming to cover the emerging domains of gestational guilt and avoiding overly complex or reverse-worded formulations, thereby producing the *initial draft of the PGAS*. In a subsequent cognitive debriefing exercise, pregnant women rated each item for clarity and relevance on a 7-point Likert scale and suggested wording changes; items with low ratings were revised or removed, which led to the *post–cognitive debriefing version of the PGAS* with adequate comprehensibility and face validity.

#### 2.3.2. Phase 2—Expert-Based Content Evaluation (CVI and Modified Kappa Criteria)

Three experts in psychometrics, perinatal psychology, and maternal health independently evaluated the *post-cognitive debriefing PGAS* by rating item relevance on a 4-point scale. Agreement on item relevance was quantified [27] using the Item-level Content Validity Index (I-CVI—proportion of experts rating 3 or 4, divided by n) and the scale-level CVI (S-CVI). Given the small panel (*n* = 3), conservative retention criteria were implemented: items required I-CVI = 1.00 and S-CVI (average I-CVI) ≥ 0.90. To control for chance agreement, the modified Kappa was also computed for each item (interpreted as: ≥ 0.75 = excellent, 0.60–0.74 = good, 0.40–0.59 = fair). Items not meeting these conservative thresholds were reworded or eliminated, resulting in the *expert-refined version of the PGAS*. Additionally, experts were given space to provide brief qualitative suggestions to improve item wording and coverage, which were used to refine the retained items.

#### 2.3.3. Phase 3—Exploratory Factor Analysis (EFA)

The factorial structure of the *expert-refined version of the PGAS* was examined using exploratory factor analysis (EFA). Assumptions of univariate normality, linearity, and homogeneity were verified via inspection of skewness (±2), kurtosis (±3), histograms, and Q-Q plots, while Mardia’s test (≤0.05 suggested deviations) assessed multivariate normality. Sampling adequacy was confirmed using the Kaiser-Meyer-Olkin (KMO) index (>0.80) and Bartlett’s test of sphericity (*p* < 0.05). To determine the optimal number of factors, and to ensure a flexible, data-driven approach [28], we combined several criteria: Horn’s parallel analysis (factors with empirical eigenvalues > random data), Cattell’s scree plot (visual inspection of the point of inflection), and Velicer’s Minimum Average Partial (MAP) test (solution minimizing average squared partial correlations). Considering the modest sample size and near-normal item distributions, EFA was conducted using Pearson correlations and MINRES extraction with Promax rotation [28,29]. Where criteria suggested different numbers of factors, we prioritized solutions supported by at least two of the three criteria and assessed their conceptual interpretability, thus driving factor-retention by joint empirical and substantive considerations. Items were considered for removal [30,31]. if communalities were below 0.40, cross-loading differences were under 0.15, or cross-load values exceeded 0.32 on two or more factors. The overall fit of the factor model was assessed using the Root Mean Square of the Residuals (RMSR ≤ 0.05) and the Fit Index (values close to 1.00 indicate good fit) [31]. The refined solution resulted in the *post-EFA version of the PGAS*.

#### 2.3.4. Phase 4—Confirmatory Factor Analysis (CFA) and Further Validation

To further assess the robustness of the factor solution under stricter model specifications, the *post-EFA version of the PGAS* was re-examined in the main sample (*n* = 171). Based on the violation of multivariate normality detected by Mardia’s test and the ordinal scaling of the items, the weighted least squares mean and variance adjusted estimator (WLSMV) was applied. As this estimator’s *χ*^2^ statistic may over-reject acceptable models with ordinal indicators and modest sample sizes [32], we relied on alternative fit indices including the Tucker–Lewis Index (TLI), the Comparative Fit Index (CFI), the Standardized Root Mean Square Residual (SRMR), and the Root Mean Square Error of Approximation (RMSEA) with its 90% confidence interval. Acceptable model fit is often defined as CFI/TLI ≥ 0.90, SRMR ≤ 0.10, and RMSEA ≤ 0.08 [33,34], whereas Hu and Bentler [35] recommend stricter criteria of CFI/TLI ≥ 0.95, SRMR ≤ 0.08, and RMSEA ≤ 0.06 to indicate good fit. The model’s local fit was evaluated through standardized factor loadings (λ) and item reliability (R^2^), with λ ≥ 0.70 and R^2^ ≥ 0.50 considered indicative of adequate item performance [31]. For internal consistency evaluation, Cronbach’s alpha (α) and McDonald’s omega (ω) were computed, with ω offering a more precise estimate when item loadings varied; values of 0.70 or higher on both indices were considered indicative of satisfactory reliability [36]. Finally, alternative structural configurations were tested, comparing the EFA-derived solution with unidimensional, bifactor, and second-order models to evaluate the best-fitting representation of the data.

Group comparisons were conducted using independent samples *t*-tests to examine potential differences in both the total PGAS score and its dimensions across relevant contextual variables (e.g., prior motherhood vs. no prior motherhood, considered pregnancy termination vs. no considered pregnancy termination). Effect sizes were estimated using Cohen’s d, with values of 0.20, 0.50, and 0.80 representing small, medium, and large effects, respectively [37].

Convergent and discriminant validity were evaluated by computing Pearson’s bivariate correlations between PGAS scores and a set of theoretically related psychological constructs, namely dispositional guilt, affect, self-esteem, perceived social support, gestational distress, and antenatal depression. Significant and sufficiently large correlations suggested convergent validity [38], while correlations below 0.90 were considered indicative of discriminant validity [36].

Incremental validity was examined using multiple linear regression analyses, testing whether PGAS dimensions explained additional variance in antenatal depression and gestational distress scores beyond sociodemographic variables, contextual factors (pregnancy stage, planned vs. unplanned pregnancy, presence of gestational complications), and other potential psychological predictors (negative affect, social support and dispositional guilt). Dichotomous covariates were dummy-coded, and all predictors were entered simultaneously using the *enter* method to assess the unique contribution of PGAS scores to the outcomes.

## 3. Results

### 3.1. Results of Phase 1—Item Generation and Comprehension Review

The thematic analysis of the two focus groups conducted with pregnant women revealed five major categories of guilt: (1) guilt related to negative thoughts or emotions, such as stress, anxiety, or a perceived lack of maternal enthusiasm; (2) guilt linked to social and family expectations, including perceived societal pressure or comparisons with other mothers; (3) guilt associated with self-care and physical-emotional well-being, such as difficulties prioritizing personal health or rest; (4) guilt related to the professional role, including concerns about returning to work or not meeting previous professional standards; and (5) guilt derived from comparisons with previous maternity experiences, such as feeling less connected or enthusiastic about the current pregnancy. The identified thematic domains, established through consensus-based coding, informed the development of the initial item pool created subsequently in this stage.

Following this, the research team generated 30 items (six per thematic domain), drawing directly from participants’ descriptions of guilt-related experiences. This process was also informed by a focused review of prior research on guilt as a psychological construct, as well as relevant literature on pregnancy and early motherhood. The initial draft of the PGAS was designed to retain the tone, complexity, and emotional nuance present in participants’ narratives, ensuring the items were both conceptually clear and culturally sensitive. Items were designed to be emotionally resonant, capturing both feelings and thoughts, and responses were recorded using a 6-point scale, from strong disagreement to strong agreement.

During the cognitive debriefing process, participants rated the 30 items for clarity and relevance using a 7-point scale. Most items obtained mean ratings above 5.2 in both aspects. However, two items were identified by several participants as being less clear, one due to ambiguity about whether the emotional content referred specifically to pregnancy-related situations, and another because it blended emotional responses with cognitive appraisals of motherhood that participants themselves perceived as distinct and independent experiences. In addition, five out of eight participants remarked that items referring to previous pregnancies were difficult to evaluate in their current context, as they were expecting their first child. Although these items were not considered confusing, their perceived lack of relevance and the limited expected representation of multiparous women in the sample prompted the research team to remove the entire fifth dimension. This dimension was removed as it reflected a history-dependent facet of guilt that would only be applicable to a subgroup of pregnant women and could distort the representation of guilt linked to the *current* pregnancy experience, given its unsuitability for robust factor analysis in later phases of the study. As a result, the *post-cognitive debriefing version* of the PGAS retained 22 items across four dimensions.

### 3.2. Results of Phase 2—Expert-Based Content Evaluation

The expert-based content evaluation of the PGAS items yielded generally high content validity indices. Each of the three experts rated the 22 items for relevance using a 4-point scale, and item-level CVI values (I-CVI) ranged from 0.33 to 1.00, and the S-CVI was 0.91, indicating adequate overall content validity for the retained item set. While the majority of items achieved full agreement among experts (I-CVI = 1.00), three received notably lower ratings, with only one of the three experts judging them as quite or highly relevant (I-CVI = 0.33), a level below the threshold for potential revision or removal [27]. Given the small panel size, we also examined modified Kappa values to adjust for chance agreement: all retained items showed a modified Kappa of 1.00, whereas the three low-CVI items showed very poor values (modified Kappa = 0.07, i.e., clearly below the 0.40 threshold for acceptable agreement). Based on this pattern of CVI and modified Kappa results, and considering the experts’ qualitative comments (limited pregnancy-specific content, conceptual blending, and overly future-oriented wording), these three items were directly removed rather than reworded. The resulting *expert-refined version of the PGAS* consisted of 19 items, which were carried forward to the exploratory factor analysis.

### 3.3. Results of Phase 3—Examination of the Underlying Factor Structure

The *expert-refined version of the PGAS* (19 items) met the assumptions for factor analysis, with a meritorious KMO value of 0.889 and a significant Bartlett’s test (χ^2^ = 1080.66; df = 171; *p* < 0.001). Excluding the previously hypothesized fifth factor (related to guilt from comparisons with previous maternity experiences, which was removed based on cognitive debriefing feedback), both scree plot and MAP test converged on the four-factor solution consistent with the thematic analysis, whereas parallel analysis suggested three. We therefore inspected both the three- and four-factor solutions. The three-factor model showed higher residual misfit and poorer structure (RMSR = 0.051 vs. 0.032; Fit Index = 0.987 vs. 0.995), with items from different content domains collapsing onto the same factor and greater overall item complexity. In contrast, the four-factor model preserved these domains and yielded a cleaner, more interpretable pattern, so we retained it as the most defensible solution. The EFA results are in the left section of Table 3. The four identified factors accounted for 63.7% of the variance, with good model fit (SRMR = 0.032; Fit Index = 0.995). However, several items displayed communalities below 0.40, cross-loadings above 0.32, or cross-loading differences under 0.15, leading to excessive item complexity (as shown in the table). Consequently, three items, belonging to different dimensions, were removed from the scale.

A revised EFA was then performed on a 16-item structure. The KMO value remained meritorious at 0.89, and Bartlett’s test was again significant (χ^2^ = 966.84; df = 120; *p* < 0.001). As before, the scree plot and MAP test indicated four factors, whereas the parallel analysis suggested three. We again compared three- and four-factor solutions. The three-factor model showed higher residual misfit and poorer fit indices (RMSR = 0.045 vs. 0.028; Fit Index = 0.991 vs. 0.997) and less clear factor boundaries, whereas the four-factor solution provided a cleaner, more interpretable structure. Given its better empirical fit and its support from two of the three retention criteria and from our theoretical framework, we retained the four-factor solution. The right section of Table 3 shows the factor loadings, communalities, and complexity for this refined solution, which exhibited a cleaner structure with higher loadings (ranging from 0.62 to 0.98), the absence of cross-loadings, and an average item complexity of 1.1. The four factors together explained 69.3% of the total variance, and the model demonstrated excellent fit (SRMR = 0.028; Fit Index = 0.997). Taken together, these results supported adopting this refined 16-item configuration as the *post-EFA version of the PGAS*.

### 3.4. Results of Phase 4—Factor Structure Replication, Reliability, Group Comparisons, and Validity Testing

**Figure 1:**
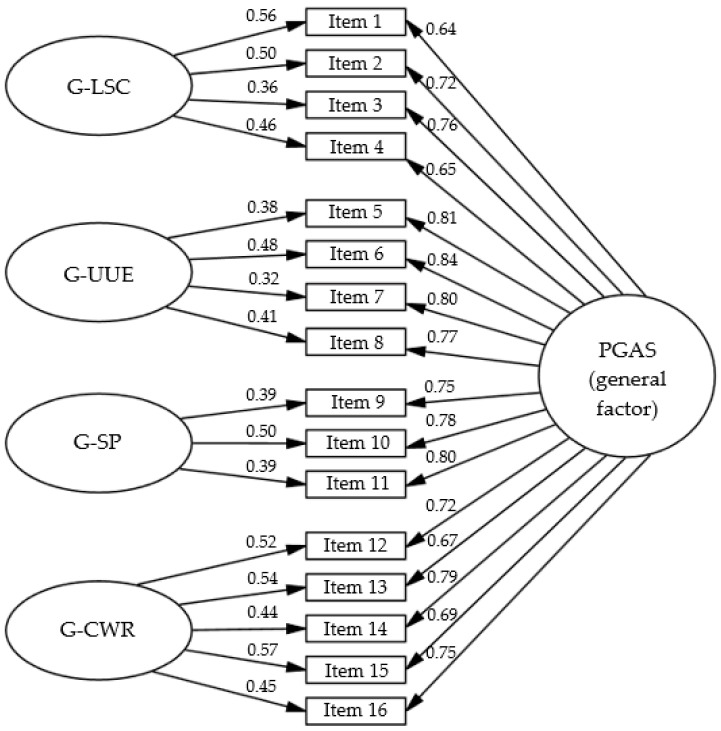
Bifactor model of the Pregnancy Guilt Assessment Scale (PGAS). Note. G-LSC = guilt related to lack of self-care; G-UEE = guilt related to unmet emotional expectations; G-SP = guilt related to social pressure; G-CWR = guilt related to conflict with work role. All coefficients shown are standardized and statistically significant (*p* < 0.001).

The CFA, conducted on the *post-EFA version of the PGAS*, compared four theoretically relevant configurations estimated with the WLSMV method: a single-factor model (discarding the sufficiency of a general factor), a correlated four-factor model (replicating the EFA outcome), a second-order four-factor model (first-order factors subsumed under a higher-order latent factor, also derived from the EFA), and a bifactor model (derived from the EFA structure, comprising one general and four specific factors). Fit indices showed that the single-factor model had clearly inadequate fit (*χ*^2^ = 682.95, df = 104, *p* < 0.001, CFI = 0.747, TLI = 0.708, RMSEA = 0.199, 90% CI [0.183–0.217], SRMR = 0.099), confirming that the construct could not be explained solely by a single latent dimension. In contrast, the correlated four-factor model showed adequate fit (*χ*^2^ = 130.85, df = 98, *p* = 0.015, CFI = 0.969, TLI = 0.962, RMSEA = 0.072, 90% CI [0.048–0.093], SRMR = 0.032), as did the second-order four-factor model (*χ*^2^ = 131.87, df = 100, *p* = 0.018, CFI = 0.969, TLI = 0.962, RMSEA = 0.072, 90% CI [0.048–0.093], SRMR = 0.034). However, the bifactor model provided the best overall fit (*χ*^2^ = 109.42, df = 88, *p* = 0.061, CFI = 0.974, TLI = 0.965, RMSEA = 0.069, 90% CI [0.044–0.092], SRMR = 0.030). While the RMSEA for all three multidimensional models fell within the acceptable range (<0.08), the CFI, TLI, and SRMR values indicated good model fit according to the criteria outlined in the Section 2.3. Given these results and the conceptual advantages of modeling both the general and the four specific dimensions of pregnancy-related guilt, the bifactor solution was retained as the final measurement model (Figure 1).

In this final bifactor solution, the four specific dimensions identified in the EFA and confirmed in the CFA were labeled according to the conceptual content of their items: *guilt over lack of self-care* (G-LSC), *guilt over unmet emotional expectations* (G-UEE), *guilt over social pressure* (G-SP), and *guilt over conflict with work role* (G-CWR). These labels, along with their corresponding acronyms, will be used in all subsequent tables and analyses to facilitate interpretation. Table 2 summarizes the wording of the PGAS items and their assignment to the four domains.

For the PGAS, bifactor reliability indices indicated excellent overall consistency. The total omega (ω) was high for the general factor (0.98) and for all four specific dimensions (G-LSC = 0.94, G-UEE = 0.90, G-SP = 0.94, G-CWR = 0.91). Hierarchical omega (ωH), which partitions the variance attributable specifically to the general or the specific factors, was 0.86 for the general factor and 0.62, 0.59, 0.74, and 0.65 for G-LSC, G-UEE, G-SP, and G-CWR, respectively. Cronbach’s alpha values, calculated using polychoric correlations, were 0.96 for the total scale and 0.90, 0.94, 0.91, and 0.94 for G-LSC, G-UEE, G-SP, and G-CWR, respectively. The explained common variance (ECV) for the general factor was 0.69, supporting a bifactor structure in which the general factor predominates.

As shown in Table 4, no statistically significant differences emerged in PGAS scores between participants with and without previous children, with small effect sizes (all *p* > 0.05; Cohen’s *d* = 0.05–0.25), suggesting minimal impact of prior motherhood on pregnancy-related guilt. In contrast, women who had considered pregnancy termination scored significantly higher across all PGAS dimensions and in the total score compared to those who had not (all *p* < 0.001; Cohen’s *d* = 0.55–1.00). These differences were moderate to large in magnitude, indicating a consistent pattern of elevated guilt levels in this subgroup.

Concerning convergent and discriminant validity, both the overall score and the individual dimensions of the PGAS showed significant correlations with the external variables (see Table 5). Specifically, higher levels of pregnancy-related guilt were associated with greater dispositional guilt, negative affect, gestational distress, and antenatal depression, alongside lower self-esteem and perceived social support, with effect sizes generally in the moderate-to-strong range. Correlations between the PGAS total score and its dimensions, as well as among the dimensions themselves, were not high enough to suggest redundancy. This pattern of associations provides empirical support for the convergent and discriminant validity of the scale.

Finally, multiple linear regression analyses were conducted to examine the incremental validity of the PGAS dimensions in predicting antepartum depression and prenatal distress (Table 6). After accounting for sociodemographic variables (step 1), contextual pregnancy-related factors (step 2), and other psychological predictors—negative affect (step 3) and perceived social support (step 4)—adding the four PGAS dimensions explained an additional 3.5% of the variance in antepartum depression (*p* < 0.001) and 14.8% in prenatal distress (*p* < 0.001). In the final models, G-UEE uniquely predicted antepartum depression (β = 0.213, *p* = 0.002), whereas G-SP uniquely predicted prenatal distress (β = 0.303, *p* < 0.001); while the association of G-UEE with prenatal distress approached, but did not reach, statistical significance (β = 0.175, *p* = 0.055). The remaining PGAS dimensions (G-LSC, G-CWR) did not exhibit unique effects. The full models accounted for 72.2% of the variance in antepartum depression and 50.0% in prenatal distress.

## 4. Discussion

This study developed and provided initial validation evidence for the PGAS, a brief measure of pregnancy-related guilt. Across four phases, we combined qualitative input from the target population with expert review and psychometric modeling to arrive at a 16-item instrument with a hierarchical structure and strong internal consistency (see Table 2 for item content and subscale assignment). Taken together, findings position the PGAS as a conceptually grounded and psychometrically robust tool for research and potential clinical applications in perinatal mental health [6,11].

Beginning with content validity, focus groups and cognitive debriefing resulted in a broad but pregnancy-specific item pool of 30 items that was grounded in participants’ language and prioritized clarity. Expert ratings then helped prune items lacking pregnancy specificity or showing conceptual blending, ensuring coverage was maintained. This staged process began with target-population input, followed by expert appraisal and, finally, empirical reduction, thus minimizing construct under- or over-representation in line with best practices in scale development [17,30].

In Phase 3, EFA revealed a preliminary four-factor structure aligned with the qualitative themes identified in earlier stages, and the removal of items with low communalities or salient cross-loadings improved the internal coherence of this solution and ensured that the retained items captured the conceptual breadth of pregnancy-related guilt without redundancy. Importantly, more parsimonious solutions with fewer factors showed poorer fit and interpretability, as they tended to collapse distinct content domains, which further supported retaining this four-factor configuration for subsequent CFA. Given the modest sample size, we treated this configuration as a potentially unstable a priori model whose main value was to provide a theoretically grounded starting point, the robustness of which was then tested in Phase 4 through CFA in the larger sample. This convergence between qualitative and quantitative evidence across phases provides construct validity support [17], showing that the factor structure suggested by the EFA can be corroborated under stricter modeling conditions.

In Phase 4, we compared theoretically plausible CFA models and found that a bifactor solution—one general factor plus four specific factors—provided the best overall fit, outperforming unidimensional, correlated four-factor, and second-order alternatives (fit indices summarized in the Results). The explained common variance for the general factor (ECV = 0.69) and ωH for the general factor (0.86) indicate a substantial common core of pregnancy-related guilt, such that the general factor accounts for most of the reliable common variance in PGAS item responses. In practical terms, this suggests that the total score can be treated as the primary indicator of overall pregnancy-related guilt level, while still leaving room for meaningful specific variance at the subscale domain. This pattern supports computing both a total score and subscale scores, with the former capturing the predominant shared liability and the latter indexing distinct facets with added clinical and theoretical value. Subscale scores should therefore be interpreted as secondary, complementary indicators that provide clinically and theoretically useful nuance over and above the dominant general factor. Reliability estimates were uniformly high for the total score and for each subscale, consistent with precise measurement at both levels. Together, these findings support the use of a hierarchical model that maximizes overall sensitivity without losing interpretable nuance by dimension.

A central contribution of this work is the articulation of four specific facets that structure pregnancy-related guilt (G-LSC, G-UEE, G-SP, and G-CWR) each grounded in both the present findings and prior evidence on the psychosocial experiences of pregnant women. G-LSC refers to guilt for not adequately following physical self-care recommendations during pregnancy, such as nutrition, exercise, or medical monitoring. Within a sociocultural context that often prioritizes the needs of the fetus over those of the mother [39], these expectations may become unrealistic and contribute to feelings of inadequacy when not met, echoing previous observations on how cultural standards of the “good pregnant woman” can elicit guilt when unmet [40]. G-UEE denotes guilt for not feeling or expressing culturally expected emotions during pregnancy, such as constant happiness, in conflict with the affective ideal of the “good mother.” While pregnancy is socially portrayed as a period of joy and happiness [41], it also entails substantial physical, relational, and emotional changes that may be challenging [42], leading some women to conceal feelings of frustration or sadness they perceive as socially unacceptable [39]. G-SP captures guilt for not following social or family advice, expectations, or traditions during pregnancy, reflecting normative pressure and internalized judgment when making decisions that deviate from what is expected. This dynamic is consistent with accounts describing how social networks and cultural narratives can amplify self-comparison and feelings of inadequacy [39,43,44]. G-CWR encompasses guilt and perceived insufficiency linked to a decline in work performance during pregnancy, alongside discomfort when prioritizing one’s own well-being, missing professional goals, or struggling with role reconciliation. Similar experiences have been reported in prior qualitative research, where Baum [45] identified the workplace as a frequent source of pregnancy-related guilt, particularly when professional and maternal roles were perceived as conflicting.

Regarding group differences, PGAS scores did not vary by whether participants had previous children, with small effects. Similar null associations have been reported for general, non–pregnancy-specific state guilt, which by definition does not capture guilt tied to the woman’s own perinatal experiences [7]. This pattern is also in line with broader perinatal affective findings showing that, once psychosocial factors are accounted for, previous childbirth often bears limited or inconsistent relations to antenatal mood [46]. By contrast, participants who had at any point considered pregnancy termination scored higher in the overall PGAS score as well as across all its dimensions. This pattern aligns with abortion-stigma frameworks indicating that merely contemplating abortion can elicit moral self-scrutiny and anticipated social judgment that amplify guilt even when no termination occurs [47], and echoes qualitative evidence showing that, even in clinical contexts (e.g., terminations for medical reasons), ambivalence, fear, and distress are frequent throughout the decision-making process, helping to explain why such scenarios may heighten the evaluative burden captured by the PGAS [48]. The prominence of the G-UEE dimension in this comparison is consistent with evidence that when women’s emotions during pregnancy do not match internalized ideals of motherhood, feelings of sadness, guilt, and worthlessness become more likely [46]. Clinically, these findings support normalizing emotional ambivalence and explicitly addressing internalized “joyful pregnancy” standards when discussing reproductive dilemmas [4,49].

Regarding convergent validity, PGAS scores were positively associated with dispositional guilt and negative affect, and negatively associated with self-esteem and positive affect. These results parallel those of Yüce-Selvi and Kantas [50], who not only observed a nearly identical correlation between their work-related guilt measure and dispositional guilt, but also documented the same pattern of associations with both negative and positive affect in a sample of mothers. Notably, positive affect was only significantly related to the first two PGAS dimensions (G-LSC and G-UEE), but not to the remaining dimensions or the total score, suggesting some specificity in this association. Thus, the moderate associations observed in the present study with dispositional guilt further support the convergent validity of the PGAS, as this reflects a related but broader affective trait. Divergent validity was supported by moderate-to-strong associations between PGAS scores and antenatal depression, prenatal distress, and negative correlations with self-esteem and perceived social support. These findings align with prior evidence showing that constructs like antenatal depression and distress [7], as well as low perceived social support [51], are affectively adjacent to guilt yet conceptually distinct. Notably, the negative correlation with social support observed in our study is consistent with Dunford and Granger’s findings, while the negative association with self-esteem and the positive associations with antenatal depression and distress align with results from Tuncer-Can et al. [7], who examined depression and anxiety as related constructs. These results provide further evidence for the construct validity of the PGAS. Finally, the pattern of correlations was generally consistent across the four dimensions of guilt (G-LSC, G-UEE, G-SP, G-CWR) as well as the total PGAS score. At the same time, intercorrelations among subscales and with the total score were not high enough to suggest redundancy, which supports discriminant validity within the broader construct of gestational guilt.

Crucially, incremental validity analyses showed that the PGAS adds unique predictive signal beyond sociodemographic/contextual factors and strong affective covariates. In the final regression models, the G-UEE dimension emerged as an independent predictor of antenatal depression, whereas G-SP significantly predicted gestational distress, after adjusting for age, education, pregnancy-related variables, negative affect, and social support. This extends prior findings on the added value of guilt-related constructs—such as those reported by Dunford and Granger [51] in relation to postpartum depression—by demonstrating that pregnancy-specific guilt predicts unique variance in antenatal depression (3.5%) and gestational distress (14%), beyond not only demographic and social variables but also major emotional predictors like negative affect. These findings suggest that guilt-related experiences constitute specific perinatal vulnerabilities, not reducible to general affective states or contextual burdens, and that their differentiated sources may hold clinical relevance. Clinically, this suggests that interventions focused on restructuring internalized emotional standards might be especially pertinent when addressing antenatal depression, while psychoeducational strategies and coping skills training related to external social mandates could prove more effective in reducing pregnancy-related distress [52,53].

The present study is not exempt from limitations. Moreover, although subjective experience is very important in psychological phenomena [54], using self-report measures is also a limitation, since these measures are more susceptible to social desirability bias. Second, the expert-based content evaluation relied on a small panel of three judges; although we used conservative retention criteria, this limited number of experts may restrict the generalizability of the content validity evidence. Third, although the scale includes four dimensions of guilt that are theoretically and empirically grounded, it is possible that additional facets—such as guilt linked to leaving behind the role of daughter or to unplanned conception—were not captured. Fourth, the sample used for the exploratory factor analysis (*n* = 85), although within the minimum thresholds suggested by Hair et al. [31], is limited and represents an important shortcoming for the generalizability of the initial structure; nevertheless, the factor solution was successfully replicated in the confirmatory factor analysis. Similarly, the sample employed for the CFA (*n* = 171) is adequate considering the characteristics of the model—absence of missing values, good factor loadings, and at least three indicators per factor—but still relatively modest, particularly for this specific target population. This also prevented us from performing a measurement invariance analysis across relevant subgroups (e.g., age, parity, education). Moreover, both quantitative phases relied on non-probabilistic online recruitment panels and yielded samples skewed toward women with higher educational and socioeconomic levels, which further limits their representativeness and points to a narrow cultural and geographical scope, being exclusively Spanish. Fifth, the cross-sectional nature of our design precludes causal interpretations, and in line with suggestions by other authors [7], longitudinal studies are warranted to further explore the origins and consequences of guilt during pregnancy. Additionally, participants with current psychiatric diagnoses were excluded to avoid confounding clinical symptomatology with gestational guilt, which further restricts the generalizability of our findings to perinatal populations with mood or anxiety disorders. Furthermore, convergent and discriminant validity were assessed only through bivariate correlations, which future studies should refine using latent and multimethod approaches. Future research should also aim to validate the scale in more diverse and larger samples, both within and beyond Spanish-speaking populations, in order to confirm the stability of its factorial structure and its cross-cultural and clinical utility.

### 4.1. Implications

From a research perspective, the results obtained support the use of the PGAS total score as the primary indicator of overall degree of pregnancy-related guilt, with the four subdimensions reflecting guilt experienced in distinct domains of the gestational context. The empirical support for the bifactor model and the high internal consistency of the scale allow the total score to be considered a general indicator of pregnancy guilt burden, useful as a cross-sectional marker in population-based or longitudinal studies. At the same time, maintaining the four subscales (G-LSC, G-UEE, G-SP, and G-CWR) facilitates a more detailed analysis of risk profiles, specific patterns of emotional distress, and differential psychological mechanisms involved in guilt during pregnancy.

In the clinical setting, the usefulness of the PGAS extends to multiple levels. First, it can be used as a screening tool to identify pregnant women with high levels of guilt, allowing for early intervention before such distress leads to more severe perinatal psychopathology. Second, the subscale scores provide relevant information for clinical formulation and individualized treatment. For example, a high score on the G-LSC may indicate a need to address beliefs about self-care, nutrition, or exercise; a high score on the G-UEE could invite exploration of internalized emotional expectations or idealizations of motherhood; the G-SP refers to the weight of social and cultural norms; and the G-CWR indicates a potential conflict between the maternal role and work performance, which can generate guilt and ambivalence. Thus, the PGAS can be integrated into perinatal assessment batteries alongside established instruments such as the Edinburgh Postnatal Depression Scale (EPDS) or the Prenatal Distress Questionnaire (PDQ), particularly in contexts where targeted interventions are planned and a dimensional approach to emotional experience during pregnancy is required [8,9].

Nonetheless, the PGAS was validated in a non-clinical Spanish sample, and there-fore its diagnostic or screening applicability remains preliminary. While findings suggest potential clinical relevance, its use for diagnostic or risk classification purposes should be considered exploratory until further validation is conducted in clinical and culturally diverse populations.

### 4.2. Future Lines of Research

There are several reasonable priorities for future research. First, it is necessary to confirm the factorial invariance of the PGAS across different trimesters of pregnancy, parity levels (primiparous vs. multiparous), and diverse sociocultural contexts, in order to ensure its cross-cultural validity and applicability in heterogeneous populations. Second, it is necessary to examine the instrument’s temporal stability, as well as its sensitivity to change following psychological interventions, with the goal of using it as a clinical outcome measure. Along these lines, it is advisable to establish normative values and percentiles by trimester, which allow for more precise interpretation of individual scores in clinical practice. Third, it will be essential to explore the longitudinal predictive validity of the PGAS, particularly its ability to anticipate relevant outcomes such as postpartum depression, the quality of prenatal and postnatal bonding, and obstetric outcomes (e.g., preterm birth, low birth weight), following the recommendations of previous studies on perinatal predictors [3,4]. Fourth, there is a need to analyze clinical cutoff functions and classification strategies based on subscale profiles, which facilitate clinical decision-making regarding referral, follow-up, or treatment. Finally, it is a priority to extend the validation of the PGAS to other languages and healthcare systems so that it can be used as a standardized tool in different healthcare settings.

## 5. Conclusions

In summary, the PGAS allows for the capture of a general burden of pregnancy guilt, while preserving four distinct facets with added explanatory and clinical value. The scale demonstrates high reliability, consistent nomological relationships, and incremental utility beyond robust affective predictors. With emerging evidence regarding its temporal stability, predictive validity, and invariance, the PGAS may become a benchmark tool for identifying, understanding, and addressing pregnancy guilt within comprehensive perinatal mental health programs.

## Figures and Tables

**Table 1:** Study phases comprising the PGAS validation process.

Phase and Approach	Objective	Procedure	Sample	Scale Items Retained Per Phase
1 (Mixed–mainly qualitative)	Item generation and comprehension review	Focus groups with pregnant women to explore guilt-related experiences, followed by research team item development and cognitive debriefing with pregnant participants.	Pregnant women (focus groups: *n* = 17; cognitive debriefing: *n* = 8)	From 30 to 22 items
2 (Mixed–mainly quantitative)	Expert-based content evaluation	Expert panel to evaluate content validity via CVI.	Experts (*n* = 3)	From 22 to 19 items
3 (Quantitative)	Underlying factor structure examination	Preliminary EFA to examine the factor structure and guide item reduction.	Pregnant women (*n* = 85)	From 19 to 16 items
4 (Quantitative)	Factor structure replication, reliability, group comparisons and validity testing	CFA, internal consistency analyses, *t*-tests, bivariate correlations, and multiple linear regression.	Pregnant women (*n* = 171)	16 items

Note. CVI, content validity index; EFA, exploratory factor analysis; CFA, confirmatory factor analysis.

**Table 2:** PGAS Items and instructions. Below you will find a series of statements about experiences that can become common in pregnancy. Please indicate how often you have felt guilty in each case during the past few weeks, using a 1–6 scale (1 = never, 6 = always). [A continuación encontrarás una serie de afirmaciones sobre experiencias que pueden llegar a ser habituales en el embarazo. Indica con qué frecuencia has sentido culpa en cada caso durante las últimas semanas, usando una escala de 1 a 6 (1 = nunca, 6 = siempre)].

	Item Wording	Factor
1.	I blame myself for not eating in a completely healthy way during my pregnancy. [*Me culpo por no alimentarme de manera completamente saludable durante mi embarazo*.]	G-LSC
2.	I feel I am not taking good enough care of my body for my baby’s well-being. [*Siento que no estoy cuidando lo suficiente mi cuerpo para el bienestar de mi bebé*.]	G-LSC
3.	I reproach myself when I do not follow all medical recommendations to the letter. [*Me reprocho cuando no sigo todas las recomendaciones médicas al pie de la letra*.]	G-LSC
4.	I reproach myself for not doing enough exercise or physical activity during pregnancy. [*Me reprocho por no hacer suficiente ejercicio o actividad física durante la gestación*.]	G-LSC
5.	I feel guilty for not enjoying every moment with my baby the way other mothers seem to. [*Siento culpa por no disfrutar cada momento con mi bebé como otras madres parecen hacerlo*.]	G-UEE
6.	I blame myself for not enjoying every moment of my pregnancy the way I am expected to. [*Me culpo por no disfrutar cada momento de mi embarazo como se espera que lo haga*.]	G-UEE
7.	I feel guilty for not always feeling happy or grateful during my pregnancy. [*Me siento culpable por no sentirme siempre feliz o agradecida durante mi embarazo*.]	G-UEE
8.	I feel responsible and guilty for not being able to manage all the emotions associated with pregnancy in a positive way. [*Me siento responsable por no poder manejar todas las emociones asociadas con el embarazo de manera positiva*.]	G-UEE
9.	I feel bad and guilty if I decide not to follow family traditions or advice about pregnancy. [*Me siento mal si decido no seguir tradiciones familiares o consejos sobre el embarazo*.]	G-SP
10.	I reproach myself when other people give their opinions about my pregnancy and I think they are right. [*Me reprocho cuando otras personas opinan sobre mi embarazo y creo que tienen razón*.]	G-SP
11.	Sometimes I feel pressured by other people’s opinions about what I should do and/or feel during my pregnancy, and I blame myself for not following their advice. [*A veces me siento presionada por las opiniones de otras personas sobre lo que debería hacer y/o sentir durante mi embarazo, y me culpo por no seguir sus consejos*.]	G-SP
12.	I blame myself for not performing at work as I did before because of my pregnancy. [*Me culpo por no rendir en el trabajo como antes debido a mi embarazo*.]	G-CWR
13.	I feel bad and guilty if I consider taking time off work for my own well-being. [*Me siento mal si considero tomar un descanso laboral por mi bienestar*.]	G-CWR
14.	I blame myself for not being able to meet all my work responsibilities because of my physical or emotional state. [*Me culpo por no poder cumplir con todas mis responsabilidades laborales debido a mi estado físico o emocional*.]	G-CWR
15.	I feel guilty when I cannot meet my work goals because of the challenges of pregnancy. [*Me siento culpable cuando no puedo cumplir con mis objetivos laborales debido a los desafíos del embarazo*.]	G-CWR
16.	Sometimes I feel guilty for not being able to balance my work responsibilities and the demands of pregnancy effectively. [*A veces me siento culpable por no poder equilibrar mis responsabilidades laborales y las demandas del embarazo de manera efectiva*.]	G-CWR

Note. The PGAS was developed and validated in Spanish. English wording is provided only as an approximate translation to facilitate comprehension. G-LSC = guilt related to lack of self-care; G-UEE = guilt related to unmet emotional expectations; G-SP = guilt related to social pressure; G-CWR = guilt related to conflict with work role.

**Table 3:** EFA rotated factor matrix: overview of 19-item and 16-item solutions (*n* = 85).

	19-Item Solution						16-Item Solution (After Removing Low-Loading or Cross-Loading)					
Item	F1 Loadings	F2 Loadings	F3 Loadings	F4 Loadings	R^2^	Complexity	F1 Loadings	F2 Loadings	F3 Loadings	F4 Loadings	R^2^	Complexity
1	**0.69**	0.03	0.24	−0.12	0.59	1.29	**0.98**	−0.13	0.06	−0.17	0.71	1.10
2	**0.66**	0.11	0.28	−0.04	0.73	1.42	**0.68**	0.11	0.17	−0.02	0.71	1.18
3	**0.58**	0.12	0.09	0.13	0.61	1.24	**0.62**	0.09	0.01	0.13	0.60	1.13
4	0.06	**0.22**	−0.05	0.14	0.11	2.09						
5	**0.67**	0.10	−0.17	0.24	0.63	1.43	**0.63**	0.12	−0.19	0.22	0.57	1.52
6	0.14	**0.83**	−0.11	0.01	0.76	1.09	−0.07	**0.89**	0.00	0.07	0.78	1.03
7	0.25	**0.80**	0.01	−0.12	0.79	1.24	0.15	**0.79**	0.04	−0.05	0.77	1.08
8	0.16	**0.75**	0.01	−0.11	0.63	1.14	0.02	**0.79**	0.08	−0.05	0.64	1.03
9	0.10	**0.88**	−0.15	−0.10	0.68	1.12	−0.09	**0.93**	−0.07	−0.02	0.71	1.03
10	−0.39	**0.75**	0.20	0.18	0.67	1.79						
11	0.17	−0.03	**0.80**	−0.05	0.70	1.10	0.13	0.03	**0.72**	−0.01	0.66	1.07
12	0.15	−0.11	**0.72**	0.12	0.69	1.20	0.10	−0.05	**0.70**	0.13	0.69	1.12
13	0.09	−0.14	**0.74**	0.08	0.61	1.13	−0.13	0.03	**0.87**	0.05	0.72	1.05
14	0.03	0.00	0.08	**0.75**	0.66	1.02	0.05	−0.02	0.01	**0.78**	0.66	1.01
15	−0.01	−0.03	0.15	**0.70**	0.61	1.10	−0.07	0.00	0.14	**0.73**	0.61	1.09
16	0.02	−0.01	0.05	**0.85**	0.79	1.01	−0.07	0.04	0.05	**0.88**	0.79	1.02
17	−0.15	0.37	**0.42**	0.03	0.38	2.25						
18	0.08	−0.04	−0.16	**0.93**	0.74	1.08	0.01	0.00	−0.14	**0.93**	0.73	1.05
19	0.01	−0.07	0.20	**0.76**	0.76	1.16	−0.01	−0.06	0.16	**0.78**	0.75	1.10
	F1	F2	F3	F4			F1	F2	F3	F4		
r with F2	0.45	-	-	-			0.66	-	-	-		
r with F3	0.42	0.52	-	-			0.58	0.34	-	-		
r with F4	0.65	0.55	0.51	-			0.63	0.50	0.65	-		
Explained variance	13%	19%	13%	19%			15%	19%	13%	23%		

Note. F1–F4, factors one to four; R^2^, communalities; r, Pearson correlation coefficient; α, Cronbach’s alpha. Loadings in bold indicate the strongest factor association for each item.

**Table 4:** Group comparisons in PGAS scores by prior motherhood and pregnancy termination consideration (*n* = 171).

	No previous children (*n* = 98)		Previous children (*n* = 73)				
	M	SD	M	SD	*t*	*p*	*d*
PGAS. G-LSC	3.18	1.38	2.85	1.33	1.6	0.111	0.25
PGAS. G-UEE	2.97	1.48	2.88	1.49	0.37	0.709	0.06
PGAS. G-SP	2.38	1.21	2.32	1.39	0.32	0.751	0.05
PGAS. G-CWR	2.73	1.43	2.64	1.42	0.44	0.663	0.07
PGAS. Total score	2.84	1.15	2.69	1.25	0.79	0.431	0.12
	Not considered pregnancy termination (*n* = 118)		Considered pregnancy termination (*n* = 53)				
	M	SD	M	SD	*t*	*p*	*d*
PGAS. G-LSC	2.81	1.3	3.55	1.36	−3.3	<0.001	−0.55
PGAS. G-UEE	2.51	1.35	3.87	1.33	−6.13	<0.001	−1.00
PGAS. G-SP ^a^	2.07	1.12	2.99	1.41	−4.2	<0.001	−0.75
PGAS. G-CWR	2.36	1.34	3.43	1.34	−4.82	<0.001	−0.79
PGAS. Total score	2.46	1.06	3.49	1.17	−5.48	<0.001	−0.94

Note. *t* = Student’s *t*-statistic; *p* = *p*-value of the *t*-statistic; ^a^ = equal variances not assumed; *d* = Cohen’s *d* effect size. G-LSC = Lack of self-care; G-UEE = Unmet emotional expectations; G-SP = Social pressure; G-CWR = Conflict with work role.

**Table 5:** Descriptive statistics and bivariate correlations (r) for PGAS scores across demographic, contextual, and psychological variables (*n* = 171).

	Scores Range	M	SD	PGAS. G-LSC	PGAS. G-UEE	PGAS. G-SP	PGAS. G-CWR	PGAS. Total Score
PGAS. G-LSC	1–6	3.04	1.36	-	0.67 ***	0.59 ***	0.57 ***	0.82 ***
PGAS. G-UEE	1–6	2.93	1.48	-	-	0.66 ***	0.65 ***	0.88 ***
PGAS. G-SP	1–6	2.36	1.29	-	-	-	0.65 ***	0.82 ***
PGAS. G-CWR	1–6	2.69	1.43	-	-	-	-	0.87 ***
PGAS. Total score	1–6	2.77	1.19	-	-	-	-	-
Age	20–45	34.25	4.98	−0.14	−0.16 *	−0.15	−0.07	−0.15
Educational level	1–7	4.99	1.10	0.25 ***	0.05	0.15	0.16 *	0.18 *
Gestational age (weeks)	1–39	21.36	10.21	−0.25 **	−0.22 **	−0.17 *	−0.33 ***	−0.17 *
Time to conception (months)	0–50	7.39	9.94	−0.04	−0.18 *	−0.06	−0.088	−0.11
Hours of sleep	4–14	7.56	1.35	−0.07	−0.11	−0.16 *	−0.08	−0.12
Perceived sleep quality	1–5	2.61	0.92	0.17 *	0.17 *	0.064	0.14	0.17 *
Planned pregnancy (no/yes)	0–1	0.73	0.45	0.03	−0.08	0.09	0.01	0.01
Pregnancy complications (no/yes)	0–1	0.19	0.40	0.22 **	0.17 *	0.17 *	0.22 **	0.23 **
Dispositional guilt	5–20	12.60	3.83	0.38 ***	0.44 ***	0.38 ***	0.39 ***	0.47 ***
Positive affect	6–25	16.61	3.92	−0.18 *	−0.17 *	−0.01	−0.05	−0.12
Negative affect	5–24	12.10	4.59	0.50 ***	0.54 ***	0.48 ***	0.43 ***	0.57 ***
Self-esteem	10–40	29.68	6.18	−0.42 ***	−0.53 ***	−0.47 ***	−0.40 ***	−0.53 ***
Perceived social support	12–42	32.80	6.84	−0.14	−0.22 **	−0.13	−0.12	−0.18 *
Antepartum depression	10–39	20.50	6.46	0.51 ***	0.61 ***	0.52 ***	0.42 ***	0.60 ***
Prenatal distress	12–50	29.77	7.58	0.55 ***	0.58 ***	0.62 ***	0.51 ***	0.65 ***

Note. G-LSC = Lack of self-care; G-UEE = Unmet emotional expectations; G-SP = Social pressure; G-CWR = Conflict with work role. Educational level (1 = primary to 7 = doctorate) and sleep quality (1 = very good to 5 = very poor) were ordinal variables; reported means indicate their relative positions on these scales. * *p* < 0.05; ** *p* < 0.01; *** *p* < 0.001.

**Table 6:** Multiple linear regression examining the independent contributions of PGAS dimensions to antepartum depression and prenatal distress (n = 171).

	Antepartum Depression				Prenatal Distress			
Predictors	*β*	*p*	Δ Adj. *R^2^*	*p*	*β*	*p*	Δ Adj. *R^2^*	*p*
Step 1			0.017	0.084			0.059	0.002
Age	−0.168	0.030			−0.096	0.202		
Educational level	0.055	0.470			0.258	<0.001		
Step 2			0.068	0.002			0.032	0.033
Gestational age	−0.218	0.004			−0.147	0.052		
Planned pregnancy	−0.057	0.446			0.035	0.639		
Pregnancy complications	−0.150	0.048			−0.136	0.070		
Step 3			0.569	<0.001			0.260	<0.001
Negative affect	0.786	<0.001			0.534	<0.001		
Step 4			0.033	<0.001			0.001	0.273
Perceived social support	−0.192	<0.001			−0.071	0.273		
Step 5			0.035	<0.001			0.148	<0.001
PGAS. G-LSC	0.004	0.952			0.061	0.453		
PGAS. G-UEE	0.213	0.002			0.175	0.055		
PGAS. G-SP	0.103	0.102			0.303	<0.001		
PGAS. G-CWR	−0.090	0.132			0.017	0.829		
Total adjusted *R^2^*			0.722				0.500	

*β* = standardized beta coefficients with *t*-test significance; Δ Adj. *R*^2^ = increment in adjusted *R*^2^ and its *F*-change significance. G-LSC = Lack of self-care; G-UEE = Unmet emotional expectations; G-SP = Social pressure; G-CWR = Conflict with work role. Predictors from earlier steps are excluded at later steps for clarity.

## Data Availability

The data presented in this study are available on request from the corresponding author. The data are not publicly available due to privacy restrictions.

## References

[1] Bjelica A., Cetkovic N., Trninic-Pjevic A., Mladenovic-Segedi L. (2018). The phenomenon of pregnancy—A psychological view. Ginekol. Pol..

[2] Catalá P., Peñacoba C., Écija C., Gutiérrez L., Meireles L.G.V. (2025). Psychological Needs in Spanish Pregnant Women During the Transition to Motherhood: A Qualitative Study. Societies.

[3] Jackson L., O’Donoghue E., Helm J., Gentilcore R., Hussain A. (2024). ‘Some Days Are Not a Good Day to Be a Mum’: Exploring Lived Experiences of Guilt and Shame in the Early Postpartum Period. Eur. J. Investig. Health Psychol. Educ..

[4] Rotkirch A., Janhunen K. (2010). Maternal Guilt. Evol. Psychol..

[5] Sutherland J. (2010). Mothering, Guilt and Shame. Sociol. Compass.

[6] Caldwell J., Meredith P., Whittingham K., Ziviani J. (2021). Shame and guilt in the postnatal period: A systematic review. J. Reprod. Infant Psychol..

[7] Tuncer-Can S., Yildiz S., Torun R., Omeroglu I., Golbasi H. (2024). Levels of anxiety, depression, self-esteem, and guilt in women with high-risk pregnancies. J. Clin. Med..

[8] Staneva A.A., Bogossian F., Morawska A., Wittkowski A. (2017). “I just feel like I am broken. I am the worst pregnant woman ever”: A qualitative exploration of the “at odds” experience of women’s antenatal distress. Health Care Women Int..

[9] Pierce S.K., Reynolds K.A., Hardman M.P., Furer P. (2022). How do prenatal people describe their experiences with anxiety? A qualitative analysis of blog content. BMC Pregnancy Childbirth.

[10] Freitas-Jesus J.V., Sánchez O.D.R., Rodrigues L., Faria-Schützer D.B., Serapilha A.A.A., Surita F.G. (2022). Stigma, guilt and motherhood: Experiences of pregnant women with COVID-19 in Brazil. Women Birth.

[11] Beck C.T. (2002). Postpartum Depression: A Metasynthesis. Qual. Health Res..

[12] Zabalegui L. (1993). Una escala para medir culpabilidad (SC-35). Miscelánea Comillas.

[13] Yali A.M., Lobel M. (1999). Coping and distress in pregnancy: An investigation of medically high risk women. J. Psychosom. Obstet. Gynecol..

[14] Caparros-Gonzalez R.A., Perra O., Alderdice F., Lynn F., Lobel M., García-García I., Peralta-Ramírez M.I. (2019). Psychometric validation of the Prenatal Distress Questionnaire (PDQ) in pregnant women in Spain. Women Health.

[15] Cox J.L., Holden J.M., Sagovsky R. (1987). Detection of Postnatal Depression. Br. J. Psychiatry.

[16] Garcia-Esteve L., Ascaso C., Ojuel J., Navarro P. (2003). Validation of the Edinburgh Postnatal Depression Scale (EPDS) in Spanish mothers. J. Affect. Disord..

[17] Boateng G.O., Neilands T.B., Frongillo E.A., Melgar-Quiñonez H.R., Young S.L. (2018). Best practices for developing and validating scales for health, social, and behavioral research: A primer. Front. Public Health.

[18] Vogt D.S., King D.W., King L.A. (2004). Focus groups in psychological assessment: Enhancing content validity by consulting members of the target population. Psychol. Assess..

[19] Morgan D.L. (2019). Basic and Advanced Focus Groups.

[20] Nyumba T.O., Wilson K., Derrick C.J., Mukherjee N. (2018). The use of focus group discussion methodology: Insights from two decades of application in conservation. Methods Ecol. Evol..

[21] Vázquez M.B., Míguez M.C. (2019). Validation of the Edinburgh postnatal depression scale as a screening tool for depression in Spanish pregnant women. J. Affect. Disord..

[22] Gargurevich R. (2010). Propiedades psicométricas de la versión internacional de la Escala de Afecto Positivo y Negativo-forma corta (I-Spanas SF) en estudiantes universitarios. Persona.

[23] Watson D., Clark L.A., Tellegen A. (1988). Development and validation of brief measures of positive and negative affect: The PANAS scales. J. Pers. Soc. Psychol..

[24] Martín-Albo J., Núñiez J.L., Navarro J.G., Grijalvo F. (2007). The Rosenberg Self-Esteem Scale: Translation and validation in university students. Span. J. Psychol..

[25] Landeta O., Calvete E. (2002). Adaptación y validación de la Escala Multidimensional de Apoyo Social Percibido. Ansiedad Estrés.

[26] Hinkin T.R. (1998). A brief tutorial on the development of measures for use in survey questionnaires. Organ. Res. Methods.

[27] Polit D.F., Beck C.T., Owen S. (2007). V Is the CVI an acceptable indicator of content validity? Appraisal and recommendations. Res. Nurs. Health.

[28] Lloret-Segura S., Ferreres-Traver A., Hernández-Baeza A., Tomás-Marco I. (2014). El análisis factorial exploratorio de los ítems: Una guía práctica, revisada y actualizada [Exploratory factor analysis of items: A practical guide, revised and updated]. An. Psicol..

[29] Fabrigar L.R., Wegener D.T., MacCallum R.C., Strahan E.J. (1999). Evaluating the use of exploratory factor analysis in psychological research. Psychol. Methods.

[30] Worthington R.L., Whittaker T.A. (2006). Scale development research: A content analysis and recommendations for best practices. Couns. Psychol..

[31] Hair J., Black W., Babin B., Anderson R. (2019). Multivariate Data Analysis.

[32] Li C.-H. (2016). Confirmatory factor analysis with ordinal data: Comparing robust maximum likelihood and diagonally weighted least squares. Behav. Res. Methods.

[33] Browne M.W., Cudeck R., Bollen K.A., Long J.S. (1993). Alternative ways of assessing model fit. Testing Structural Equation Models.

[34] Bentler P.M. (1990). Comparative fit indexes in structural models. Psychol. Bull..

[35] Hu L., Bentler P.M. (1999). Cutoff criteria for fit indexes in covariance structure analysis: Conventional criteria versus new alternatives. Struct. Equ. Model. A Multidiscip. J..

[36] Kline R.B. (2011). Principles and Practice of Structural Equation Modeling.

[37] Cohen J. (1988). Statistical Power Analysis for the Behavioral Sciences.

[38] Campbell D.T., Fiske D.W. (1959). Convergent and discriminant validation by the multitrait-multimethod matrix. Psychol. Bull..

[39] Lupton D. (2017). ‘It just gives me a bit of peace of mind’: Australian women’s use of digital media for pregnancy and early motherhood. Societies.

[40] Facca D., Hall J., Hiebert B., Donelle L. (2023). Understanding the tensions of “good motherhood” through women’s digital technology use: Descriptive qualitative study. JMIR Pediatr. Parent..

[41] Suppes A. (2020). Do women need to have children in order to be fulfilled? A system justification account of the motherhood norm. Soc. Psychol. Personal. Sci..

[42] Al-Mutawtah M., Campbell E., Kubis H.-P., Erjavec M. (2023). Women’s experiences of social support during pregnancy: A qualitative systematic review. BMC Pregnancy Childbirth.

[43] Tate M.K. (2023). The impact of social comparison via social media on maternal mental health, within the context of the intensive mothering ideology: A scoping review of the literature. Issues Ment. Health Nurs..

[44] Tosun H., Özkan H. (2025). Problematic social media use and its relationship with breastfeeding behaviors and anxiety in social media-native mothers: A mixed-methods study. Healthcare.

[45] Baum N. (2006). Pregnant field student’s guilt. J. Soc. Work Educ..

[46] Biaggi A., Conroy S., Pawlby S., Pariante C.M. (2016). Identifying the women at risk of antenatal anxiety and depression: A systematic review. J. Affect. Disord..

[47] Norris A., Bessett D., Steinberg J.R., Kavanaugh M.L., De Zordo S., Becker D. (2011). Abortion stigma: A reconceptualization of constituents, causes, and consequences. Women’s Health Issues.

[48] Sereno S., Leal I., Maroco J. (2013). The role of psychological adjustment in the decision-making process for voluntary termination of pregnancy. J. Reprod. Infertil..

[49] Martín-Sánchez M.B., Martínez-Borba V., Catalá P., Osma J., Peñacoba-Puente C., Suso-Ribera C. (2022). Development and psychometric properties of the maternal ambivalence scale in spanish women. BMC Pregnancy Childbirth.

[50] Yüce-Selvi Ü., Kantaş Ö. (2019). The psychometric evaluation of the maternal employment guilt scale: A development and validation study. Isg. J. Ind. Relat. Hum. Resour..

[51] Dunford E., Granger C. (2017). Maternal guilt and shame: Relationship to postnatal depression and attitudes towards help-seeking. J. Child Fam. Stud..

[52] Sockol L.E. (2018). A systematic review and meta-analysis of interpersonal psychotherapy for perinatal women. J. Affect. Disord..

[53] Milgrom J., Gemmill A.W. (2014). Screening for perinatal depression. Best Pract. Res. Clin. Obstet. Gynaecol..

[54] Robinson M.E., Staud R., Price D.D. (2013). Pain Measurement and Brain Activity: Will Neuroimages Replace Pain Ratings?. J. Pain.

